# Effects of *Tabernaemontana elegans* and *Lantana camara-derived nanoparticles* on the hatching and mortality of *Meloidogyne incognita* second-stage juveniles under *in vitro* conditions

**DOI:** 10.3389/fpls.2026.1779007

**Published:** 2026-03-20

**Authors:** Nicholus M. Mnyambo, Zakheleni P. Dube, Nokuthula Khanyile

**Affiliations:** Faculty of Agriculture and Natural Sciences, University of Mpumalanga, Mbombela, South Africa

**Keywords:** aloe vera, *Lantana camara*, Meloidogyne incognita, nanotechnology, *Tabernaemontana elegans*

## Abstract

This study investigated the synthesis of green nanoparticles (NPs) from *Lantana camara* (NPlc) and *Tabernaemontana elegans* (NPte) using *Aloe vera* as a reducing and stabilizing agent. It further assessed the impact of the synthesized nanoparticles on *Meloidogyne incognita* juvenile hatch and mortality. Nanoparticles were synthesized by combining plant extracts and *A. vera* gel in five volume ratios (100:0, 25:75, 50:50, 75:25, and 0:100 v/v). The physical characteristics of the nanoparticles were analyzed using UV-Vis spectroscopy, transmission electron microscopy (TEM), and selected area electron diffraction (SAED). The results confirmed the polycrystalline structure and morphological diversity (cubic, triangular, platelet, and irregular forms) of the nanoparticles, with composition ratios significantly influencing their structural properties. All concentrations with Aloe gel produced nano-scale (<21 nm) products, whereas the single extracts were larger than the accepted nano-scale size. The optimal combination of *A. vera* extract ratio was found to be 50:50, accompanied by small nanoparticles exhibiting a highly defined ring pattern and well-developed polycrystalline structures. In bioassays, different concentrations of NPlc and NPte significantly (P ≤ 0.05) inhibited *M. incognita* juvenile hatch and increased juvenile mortality. The lowest juvenile hatch rates (6.9–7%) and highest inhibition levels (72–74%) were recorded at the 50:50 plant extract to *A. vera* ratio. Hatch inhibition and mortality were time-dependent, with minimal effects observed at 24 hours and the highest (79%) responses at 72 hours of exposure. These findings demonstrate that green-synthesized nanoparticles can be produced from *L. camara* and *T. elegans*, which possess strong nematicidal potential and may serve as sustainable, eco-friendly alternatives to conventional chemical nematicides.

## 1 Introduction

Plant‐parasitic nematodes (PPNs), particularly root-knot species (*Meloidogyne* spp.), inflict multi-billion-rand losses on food and fiber crops each year and are ranked among the top plant pests of economic concern in the world (Kumar et al., 2020). Chemical nematicides, such as organophosphates and fumigants, can limit outbreaks; however, tight regulations, high costs, and documented risks to human health, beneficial soil biota, and groundwater have accelerated the need for safer alternatives (Zhou et al., 2024). The use of botanical nematicides has gained popularity in the past two decades as an alternative to synthetic nematicides.

Botanicals occupy a strategic niche in nematode management, as they combine biodegradability with complex modes of action that slow the evolution of resistance (Rocha et al., 2022). Among the plants with promising nematicidal potential are *Tabernaemontana elegans* (*Apocynaceae)* and *Lantana camara* (*Verbenaceae)* (Khosa et al., 2020; Malahlela et al., 2021). Previous studies have highlighted the efficacy of *T. elegans* in reducing nematode populations, with reports indicating a reduction of up to 97% in *M. incognita* eggs and juveniles (Malahlela et al., 2021). Similarly, *L. camara*, rich in allelochemicals, has been observed to be capable of reducing nematode-induced root galls and egg masses in infested plants (Hasan et al., 2021). Despite these findings, crude extracts face several critical limitations: their active compounds often degrade rapidly under field conditions, exhibit poor water solubility, and vary markedly in potency from season to season, necessitating frequent reapplications that lead to inconsistent nematode control (Malahlela et al., 2021). These drawbacks not only inflate operational costs but also erode farmers’ confidence in botanical nematicides (Khosa et al., 2020).

Nanotechnology offers promising solutions to enhance the efficacy of botanical pesticides and nematicides (Abd-Elgawad, 2024a). Nanoparticles have a high surface area-to-volume ratio, which enhances their biological reactivity and enables the efficient delivery of active compounds to target organisms (Yusuf et al., 2023). Beyond production, understanding how green NPs debilitate nematodes is crucial for rational design. Recent studies reveal three complementary mechanisms: (i) electrostatic adhesion and cuticle disruption, where the positively charged NPs bind to the negatively charged nematode cuticle, causing ultrastructural fissures and loss of turgor (Gautam et al., 2025); (ii) reactive-oxygen-species (ROS) generation, where the NP contact stimulates intracellular ROS (Canaparo et al., 2020; Horie and Tabei, 2021), triggering oxidative damage and apoptosis in juveniles and eggs; and (iii) sustained release of phytochemicals, which maintains lethal concentrations in the rhizosphere longer than bulk extracts (Horie and Tabei, 2021). Efficacy is strongly size- and shape-dependent, with larger agglomerates (>300 nm) primarily acting as slow-release reservoirs (Mikušová and Mikuš, 2021).

In nematode management, nanotechnology has demonstrated potential in improving the efficacy of agrochemicals, reducing the required dosages, and minimizing negative non-target effects (Abd-Elgawad, 2024b). Conventional nanoparticle (NP) syntheses, however, often rely on metal salts and synthetic reducers/stabilizers (e.g., NaBH_4_, CTAB, PVP) that leave toxic residues and whose long-term ecological footprint in agroecosystems remains poorly understood. Sudhimon et al. (2024) reported the synthesis of silver nanoparticles solely from *A. vera* gel, with particle sizes ranging from 10 to 50 nm and exhibiting significant antimicrobial and antioxidant properties. Kalaiselvi et al. (2017) produced silver nanoparticles using latex from *Euphorbia tirucalli* to control *M. incognita*, achieving 96–100% juvenile (J2) mortality within 6 hours, a 46.95% reduction in infectivity at 100 ng/mL, and complete inhibition at 1000 ng/mL under laboratory conditions. Similarly, Nasr et al. (2025) found that silver nanoparticles derived from *Citrus limon* L. caused 100% mortality of *M. javanica* J2 at 100 ppm, while also reducing disease severity by 66.67% and decreasing nematode populations by 99.27% in faba beans.

Although phyto-mediated green synthesis shows promising results in mitigating the potential ecological risks of nanoparticles, it is not free from drawbacks. For example, a study by Punniyakotti et al. (2024) reported that silver nanoparticles (AgNPs) negatively affected soil microbial biomass, while copper nanoparticles inhibited plant root elongation and had a negative impact on soil enzyme activities. Interestingly, adverse impacts were also observed with silver nanoparticles (AgNPs) synthesized using *L. camara* leaf extracts, which demonstrated antioxidants, antibacterial, and cytotoxic properties (Shriniwas and Subhash, 2017). These risks highlight the need for safer and more sustainable approaches to nanoparticle synthesis. While most previous reports primarily focused on metallic nanoparticles, the present study is distinct in that it demonstrates the formation of phytochemical-derived organic nanoparticles from *T. elegans* and *L. camara* mediated by *A. vera* gel.

The green synthesis of nanoparticles using plant extracts as reducing and stabilizing agents is a promising approach, combining safety advantages with enhanced efficacy properties (Singh et al., 2023). The effectiveness of biological or green-synthesized nanoparticles in suppressing plant-parasitic nematodes (PPNs) was demonstrated by Manimegalai et al. (2020), offering an environmentally sustainable alternative to conventional nematicides. *Aloe vera* gel is especially attractive due to its rich phytochemical profile, which includes polysaccharides, flavonoids, and polyphenols, simultaneously reducing metal ions (or phytochemical quinones) and sterically stabilizing nascent particles (Uddin et al., 2020). It contains powerful antioxidants and electron-rich compounds, such as flavonoids, phenolic acids, tannins, saponins, alkaloids, vitamins (notably vitamin C and E), enzymes (like oxidases and peroxidases), and polysaccharides (Manimegalai et al., 2020). These compounds donate electrons, breaking down larger molecules in the bulk extract and reducing them to nanoscale particles (Manimegalai et al., 2020). Simultaneously, *A. vera* polysaccharides and proteins act as natural capping agents, stabilizing the newly formed nanoparticles and preventing aggregation (Dharajiya et al., 2017). This method not only enhances the efficiency of nematicidal plant extracts but also yields a biocompatible, eco-friendly, and sustainable nanoproduct, making it ideal for agricultural applications. Using *A. vera* in this way leverages both its chemical activity and its compatibility with plant-based systems, making it a superior alternative to conventional chemical synthesis.

While previous studies primarily focused on metallic nanoparticles, the present study is distinct in that it demonstrates the formation of phytochemical-derived organic nanoparticles from *T. elegans* and *L. camara* mediated by *A. vera* gel. Despite the proven bioactivity of *T. elegans* and *L. camara* extracts, no study has systematically nano-formulated these botanicals via an *A. vera* mediated route. Limited studies have mapped how the synthesis ratio influences NP morphology, crystallinity, and optical signatures, or link these structural attributes to nematicidal performance and the mechanisms of action. Addressing these gaps is essential for translating green nano-nematicides from concept to field application. Hence, the objective of the current study was to develop a protocol for synthesizing nanoparticles of *T. elegans* and *L. camara* using *A. vera* gel as both a reducing and stabilizing agent, and to test them on *M. incognita* juvenile hatch and mortality.

## 2 Materials and methods

### 2.1 Description of the study

The research was conducted under controlled laboratory conditions at a temperature of 25 ± 2 °C and 55% relative humidity at the University of Mpumalanga, South Africa, located at latitude 25.4365°S and longitude 30.9818°E.

### 2.2 Collection and preparation of plant material

Leaves of *T. elegans* and *L. camara* were collected from Nkomazi Municipality, Mpumalanga, South Africa. The freshly harvested leaves were washed with distilled water to remove debris and contaminants. The leaves were then cut into 5 cm-long sections and air-dried at room temperature for 24 hours to remove surface moisture before further drying in an oven. Subsequently, the leaves were dried in a hot air oven set at 52 °C for four days to ensure complete moisture removal. The dried leaves were ground into a fine powder using a high-speed blender, operating for 3 minutes to achieve a uniform particle size. The powdered leaf meals were stored in light-proof, temperature-controlled (4 °C), labelled glass jars to preserve their phytochemical integrity until required.

Fresh *A. vera* gel preparation: Fresh leaves were harvested from the University of Mpumalanga, Mbombela campus. The leaves were thoroughly washed with distilled water to remove contaminants and microorganisms. The outer layer of the leaves was peeled off using a sterilized knife to extract the inner gel, which was collected in labelled sterile containers. To prepare an aqueous extract, the gel was blended with distilled water (10 g of gel to 100 ml of water) to form a colloidal solution. The resulting mixture was filtered through Whatman No. 42 filter paper and stored at 4 °C until required.

### 2.3 Preparation of aqueous extracts from plant powders

Aqueous extracts were prepared by mixing 1 g of powdered plant material with 10 mL of distilled water in 100 mL conical flasks. The mixture was agitated on a rotary shaker at 150 rpm for 48 hours at room temperature (Kalaiselvi et al., 2017). The resulting solutions were filtered and stored at 4 °C for subsequent analysis.

### 2.4 Green synthesis of nanoparticles using A. vera *gel*

Nanoparticles were synthesized by mixing five ratios [100:0, 25:75, 50:50, 75:25, and 0:100 (plant extract: *A. vera*) v/v] of each plant extract with *A. vera* gel. The ratios were chosen to systematically evaluate how varying the proportion of *A. vera* relative to the other plant extract influences nanoparticle synthesis. These ratios provide a gradient from low to high *A. vera* content, enabling assessment of its effect on the size, shape, and stability of the nanoparticles. The mixtures were stirred for 12 hours at room temperature using a magnetic stirrer. All procedures were performed under aseptic conditions, with triplicates for each ratio to ensure reproducibility.

### 2.5 Characterization of synthesized nanoparticles

#### 2.5.1 Transmission electron microscopy

Nanoparticle morphology was analyzed using a TEM (JEM-2100, Joel, Japan) operated at 200 kV. Samples were prepared by drop-casting onto holey carbon-coated copper grids and drying overnight in a desiccator. Particle size distribution was determined using ImageJ software (NIH, USA). Origin Lab 2024 software was used to plot the histogram graphs.

#### 2.5.2 Selected area electron diffraction patterns

Selected Area Electron Diffraction (SAED) is a critical technique used to analyze the crystallographic properties of nanoparticles during their formation. A small drop of this suspension was deposited onto a TEM grid made of copper and coated with a thin carbon film to provide mechanical stability. Excess liquid was removed using filter paper, and the sample was left to dry at room temperature. This preparation ensures that nanoparticles are well-dispersed on the grid, minimizing agglomeration and overlap. The SAED patterns obtained were analyzed to determine the crystallographic properties of the nanoparticles. The diffraction spots or rings correspond to specific lattice planes, and their spacing (d-spacing) was calculated using Bragg’s Law. The radius of each ring in the diffraction pattern was measured and correlated with the constant camera, which is determined during the instrument calibration. Using these measurements, the lattice parameters and crystalline structure of the nanoparticles are inferred. To ensure reliability, the SAED measurements were repeated for multiple regions on the sample grid and for different samples prepared under the same conditions. Consistency in the diffraction patterns validates the reproducibility of the nanoparticle synthesis process and the SAED measurement.

#### 2.5.3 Visual observation and UV-Vis spectra analysis

UV-Vis spectroscopy was performed using a PerkinElmer Lambda 265 spectrophotometer (PerkinElmer, Shimadzu, Japan). Spectra were recorded between 200 and 700 nm using a 1 cm pathlength quartz cuvette, with distilled water as the blank. Data smoothing was performed using the Savitsky-Golay method in OriginLab software.

### 2.6 Bioassay preparation

#### 2.6.1 Preparation of nematode inoculum

A population of *Meloidogyne incognita* race 2, identified through sequence-characterized amplified regions polymerase chain reaction (SCAR-PCR), was sourced from the ARC-Grain Crops Institute in Potchefstroom, South Africa. This population was propagated for two months in a greenhouse using the susceptible tomato cultivar ‘Star 9009’. Egg masses collected from infected tomato plants were agitated in a 1% sodium hypochlorite (NaOCl) solution for 30 seconds to dissolve the gelatinous matrix surrounding the eggs while simultaneously surface-sterilizing them (Smalley, 1988). The eggs were thoroughly rinsed with distilled water before use in the J2 hatch assays. Freshly hatched J2 were obtained by placing surface-sterilized eggs in Petri dishes containing 10 mL of distilled water and incubating them at 25 ± 2 °C for 24 hours. Concentrations (100:0, 25:75, 50:50, 75:25, and 0:100 (plant extract: *A. vera*) v/v) prepared previously were tested for J2 hatch inhibition in Petri dishes at room temperature.

#### 2.6.2 *Meloidogyne incognita* second-stage juveniles hatch inhibition bioassay

The hatch inhibition tests were conducted under *in vitro* conditions. Concentrations prepared previously were tested for J2 hatch inhibition in Petri dishes at room temperature. Three replications were performed per treatment. Egg masses were dislodged from roots into a Petri dish containing distilled water using a sterilized sharp needle. Each Petri dish was filled with 2 mL of distilled water containing 100 eggs + 8 mL of respective nanoparticles. Cumulative numbers of J2 hatch inhibition were recorded at 24, 48, and 72 hours of exposure. Leaf extracts without *A. vera* and sterilized distilled water (SDW) were also maintained as controls. Concentrations (100:0, 25:75, 50:50, 75:25, and 0:100 (plant extract: *A. vera*) v/v) were considered J2 hatch-inhibitive when the second stage hatch inhibition percentage was significantly lower than in the control. In all trials, three independent experiments with treatments replicated three times were conducted.


$$Second-stage\;juvenile\;hatch\;inhibition\;\left(\%\right)=\;\frac{\text{Total number of eggs}-\text{Number of J}2\text{ hatched}}{\text{Total number of eggs}}\;\times 100$$

#### 2.6.3 Nanoparticles on *Meloidogyne incognita* second-stage juvenile mortality assay

The mortality assays were conducted under *in-vitro* conditions, the same way as with J2 hatch inhibition, except that approximately 100 *M. incognita* J2 were added to Petri dishes instead of eggs. The J2 were considered dead if they were not mobile after being transferred into distilled water for 30 seconds, even when probed with needles and counted using a stereomicroscope. Concentrations (100:0, 25:75, 50:50, 75:25, and 0:100 (plant extract: *A. vera*) v/v) were considered J2 mortal when the second stage mortality percentage was significantly more than in the control. In all trials, three independent experiments with treatments replicated three times were conducted. The percentage of J2 mortality was calculated using the formula given below:


$$\text{Mortality }\left(\%\right)=\;\frac{\text{Number of dead juveniles mortality J}2\text{ in the treatment }}{Total\;number\;of\;J2}\;\times \;100$$

#### 2.6.4 Data analysis

Nematode data were subjected to analysis of variance (ANOVA) through Statistix 10 software. Before conducting ANOVA, the Shapiro-Wilk normality test was applied to assess data homogeneity. Non-continuous data were transformed using log_10_(x + 1) to achieve homogeneous variance. For variables showing significant differences, mean separation was performed using Fisher’s least significant difference (LSD) test at a 0.05 significance level.

## 3 Results

### 3.1 Nanoparticle synthesis

The characterization of the synthesized nanoparticles from plant extracts revealed distinct differences in particle size, shape, crystallinity, and optical behavior. The observed trends varied with the type of plant extract used and the concentration, a consistency observed across all analytical techniques: TEM, SAED, and UV-Vis spectroscopy.

#### 3.1.1 Transmission electron microscopy

**Figure 1:**
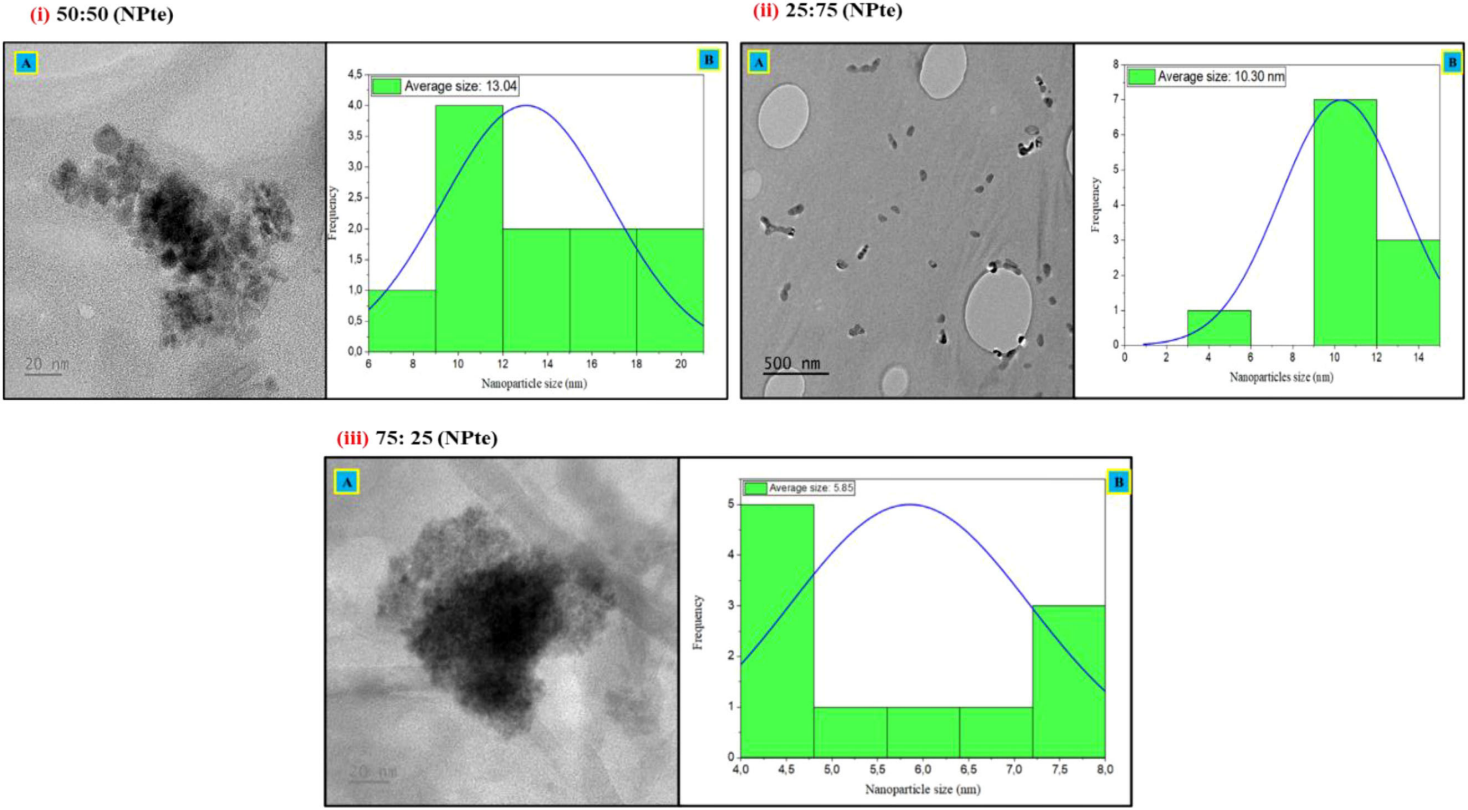
Transmission electron microscopy (TEM) images displaying *T. elegans* nanoparticles (NPte) at: (i) 50:50 ratio of *T. elegans* to *A. vera*, captured at magnifications of 20 nm **(A)** with particle size distributions curve **(B)** of NPte with an average of 13.04 nm; (ii) 25:75 ratio of *T. elegans* to *A. vera*, captured at magnifications of 500 nm **(A)** with particle size distributions curve **(B)** of NPte with an average of 10.30 nm; (iii) 75:25 ratio of *T. elegans* to *A. vera*, captured at magnifications of 20 nm **(A)** with particle size distributions curve **(B)** of NPte with an average of 5.85 nm.

**Figure 2:**
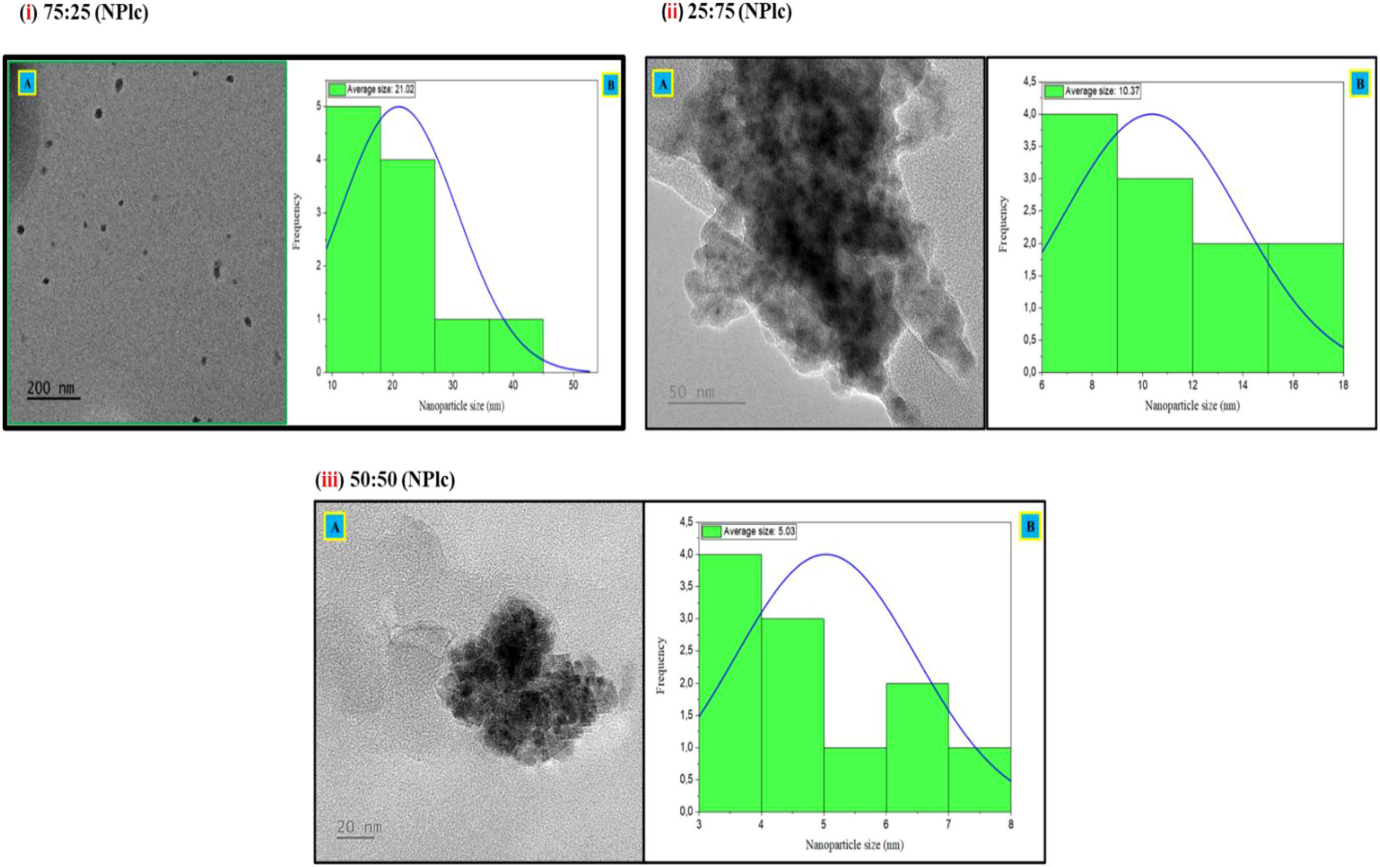
Transmission electron microscopy (TEM) images displaying *L. camara* nanoparticles (NPlc) at: (i) 75:25 ratio of *L. camara* to *A. vera* captured at magnifications of 200 nm **(A)** with average particle size distributions curve **(B)** of NPlc with an average of 21.02 nm; (ii) 25:75 ratio of *L. camara* to *A. vera* captured at magnifications of 50 nm **(A)** with average particle size distributions curve **(B)** of 10.37 nm; (iii) 50:50 ratio of *L. camara* to *A. vera* captured at magnifications of 20 nm **(A)** with average particle size distributions curve **(B)** of 5.03 nm.

**Figure 3:**
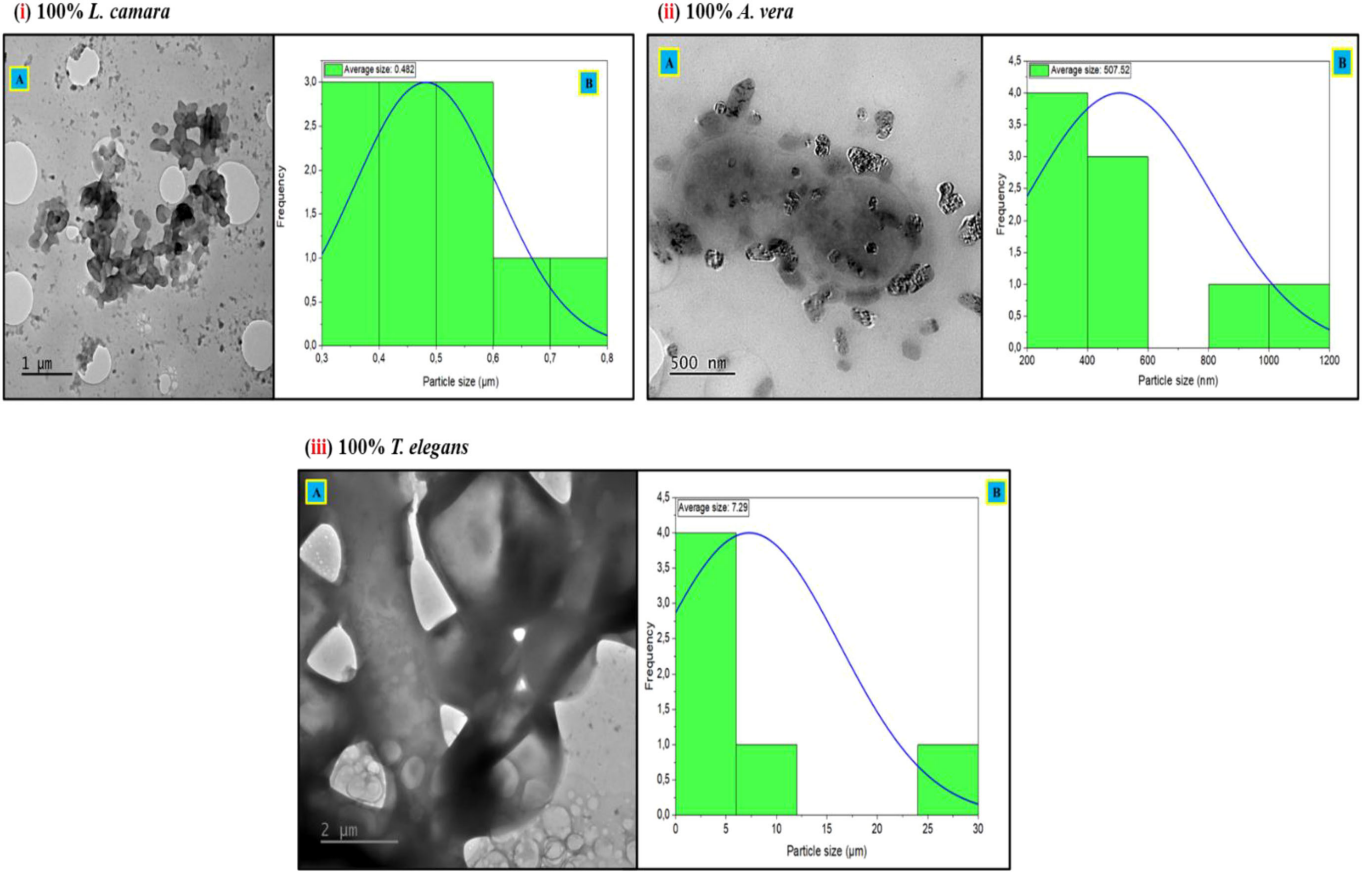
TEM images of (i) 100% *L. camara* captured at magnifications of 1 µm **(A)** with average particle size distributions curve **(B)** of 0.482 µm (482 nm); (ii) 100% *A. vera* captured at magnifications of 500 nm **(A)** with average particle size distributions curve **(B)** of 507.52 nm; (iii) 100% *T. elegans* captured at magnifications of 500 nm **(A)** with particle size distributions curve **(B)** of 7.29 µm (7290 nm).

The TEM analysis provided a detailed visualization of the structural and morphological properties of the synthesized nanoparticles. Nanoparticles derived from *T. elegans* (NPte) and *L. camara* (NPlc) exhibited varied shapes, including cubic, triangular, platelet, and irregular forms (**Figures 1**–**3**). For NPte, particle size varies with the concentration ratios of the plant extracts (**Figures 1**–**3**). The 50:50 concentration exhibited the largest particle size, averaging 13.04 nm, followed by the 25:75 concentration at 10.30 nm, while the 75:25 concentration produced the smallest average particle size of 5.85 nm (**Figure 1**).

For NPlc, the particle sizes followed a similar trend, with the 75:25 concentration producing the largest average particle size at 21.02 nm, followed by the 25:75 concentration at 10.37 nm, and the smallest average particle size of 5.03 nm, observed in the 50:50 concentration (**Figure 2**).

The single extracts from the three plants (*L. camara*, *T. elegans*, and *A. vera*) exhibited particle sizes that are outside the nanoscale of 1 to 100 nm. Particle sizes from *L. camara* and *A. vera* were 482 nm and 507.52 nm, respectively, with particle size from *T. elegans* being notably the largest, averaging 7290 nm (**Figure 3**).

### 3.2 Selected area electron diffraction patterns

**Figure 4:**
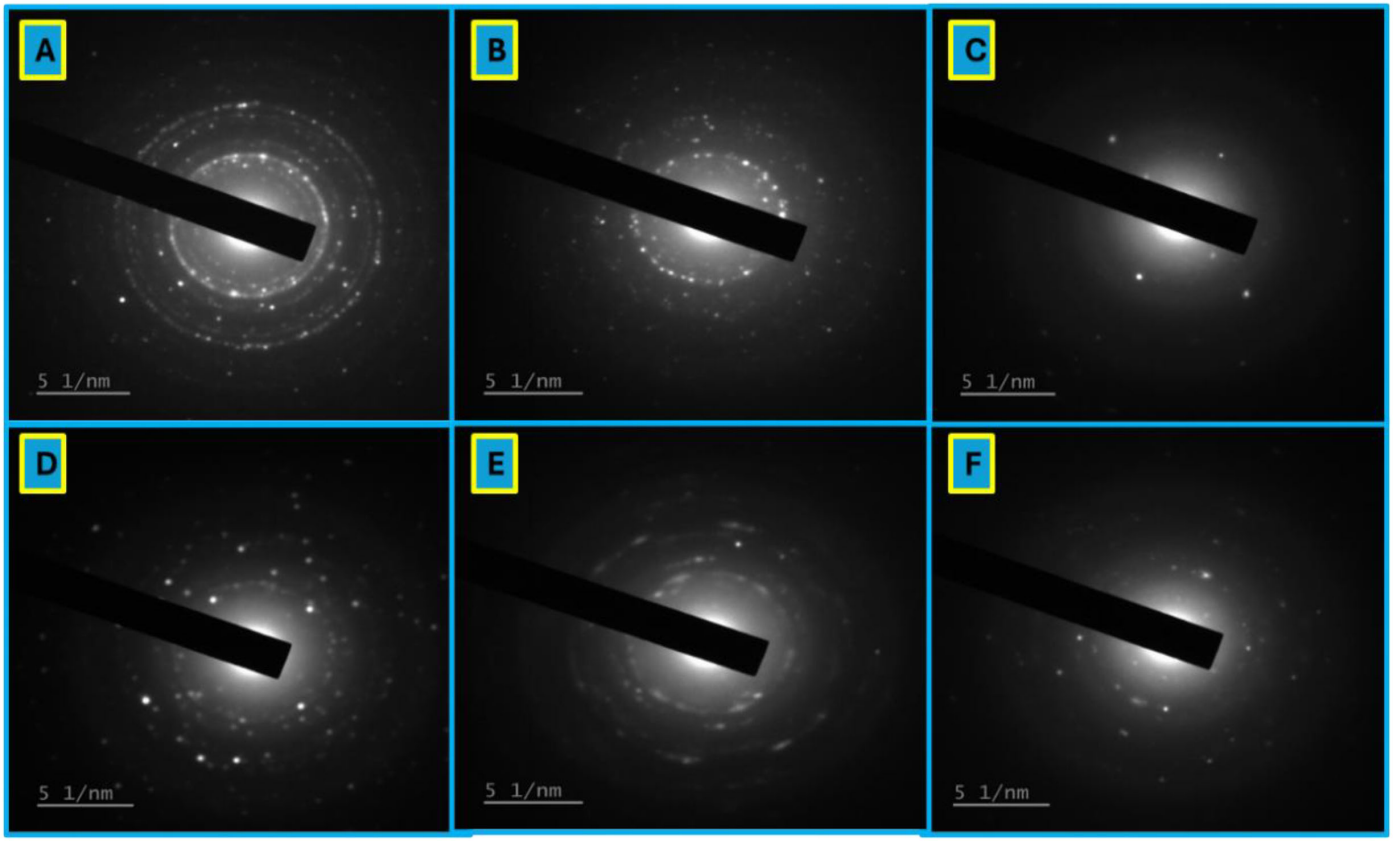
Ring SAED pattern of polycrystalline extract: *A. vera*: **(A)** 50:50 NPlc; **(B)** 25:75 NPlc; **(C)** 75:25 NPlc; **(D)** 50:50 NPte; **(E)** 25:75 NPte; **(F)** 75:25 NPte.

The Selected Area Electron Diffraction (SAED) patterns confirmed the polycrystalline nature of the synthesized nanoparticles, indicating their structural stability and crystallinity. The SAED analysis revealed that the ratio of plant extracts used during synthesis played a critical role in determining the structural characteristics of the nanoparticles (**Figure 4**). For the NPlc samples, the 50:50 ratio exhibited a highly defined ring pattern with bright and distinct spots, indicative of well-developed polycrystalline structures (**Figure 4**). However, at the lower proportion of *A. vera* (75:25), the ring patterns became less defined with reduced sharpness and uniformity (**Figure 4**). A similar trend was observed in the NPte samples. The 50:50 ratio of NPte and NPlc displayed sharp and bright SAED patterns.

#### 3.1.2 UV-Vis spectroscopy

**Figure 5:**
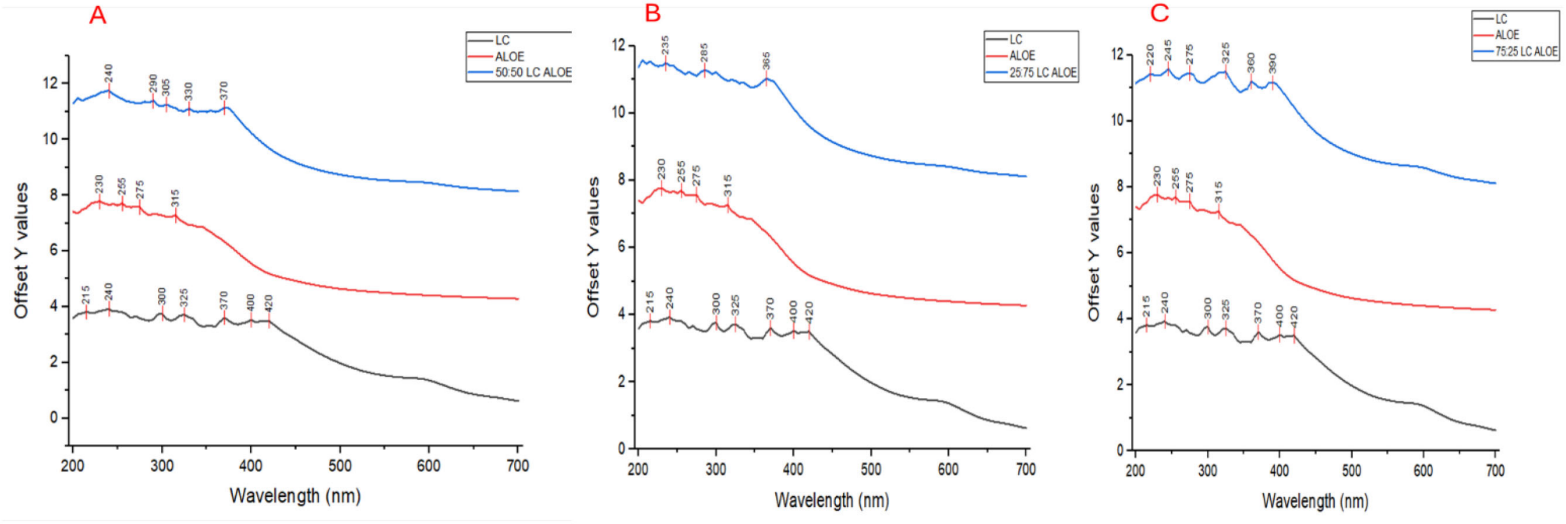
UV-Vis spectra of *Lantana camara*, *Aloe vera*, and synthesized NPlc at: **(A)** 50:50, **(B)** 25:75, and **(C)** 75:25.

The UV-Vis spectra illustrate distinct absorbance trends and spectral shifts among the *L. camara* extract (LC), *A. vera* extract (Aloe), and the nanoparticle (50:50) (LC: Aloe) (**Figure 5**). Each sample exhibits characteristic peaks, reflecting its unique chemical composition and interaction with UV light. The *L. camara* extract displays peaks at 215 nm, 240 nm, and 300 nm, with smaller peaks at 325 nm and 370 nm. The intensity of absorbance was moderate across the spectrum (**Figure 5**).

The *A. vera* extract had peaks at 230 nm, 255 nm, and 275 nm, with additional peaks at 315 nm and 370 nm (**Figure 5**). Compared to *L. camara*, the peaks for *A. vera* appear at slightly longer wavelengths, and absorbance intensities were higher than those of *L. camara*. In the 50:50 NPlc, peaks appear at 215 nm, 240 nm, 300 nm, 325 nm, and 370 nm, with additional broader peaks at 400 nm and 420 nm (**Figure 5**). The presence of broader and more intense peaks, especially in the visible region (400–420 nm). The nanoparticle spectrum also shows peak shifts and overlaps between the individual extracts (**Figure 5**). When comparing the three spectra, NPlc exhibits higher overall absorbance and broader peaks, especially in the UV-visible range.

For the 25:75 (LC: Aloe) nanoparticle (NPlc), the spectrum exhibits peaks at 235 nm, 285 nm, and 365 nm, with broader peaks in the visible region at approximately 400 nm and 420 nm (**Figure 5**). Compared to the individual extracts, the peaks have shifted and broadened in intensity. The absorbance intensity is the highest among the three samples, particularly in the UV-visible range (**Figure 5**).

The 75:25 (LC: Aloe) synthesized nanoparticle shows both shifts in the positions of existing peaks and the appearance of new peaks (**Figure 5**). The peaks observed in the *L. camara* and *A. vera* extracts are slightly shifted, suggesting an interaction between the biomolecules from both extracts during the nanoparticle formation process (**Figure 5**).

**Figure 6:**
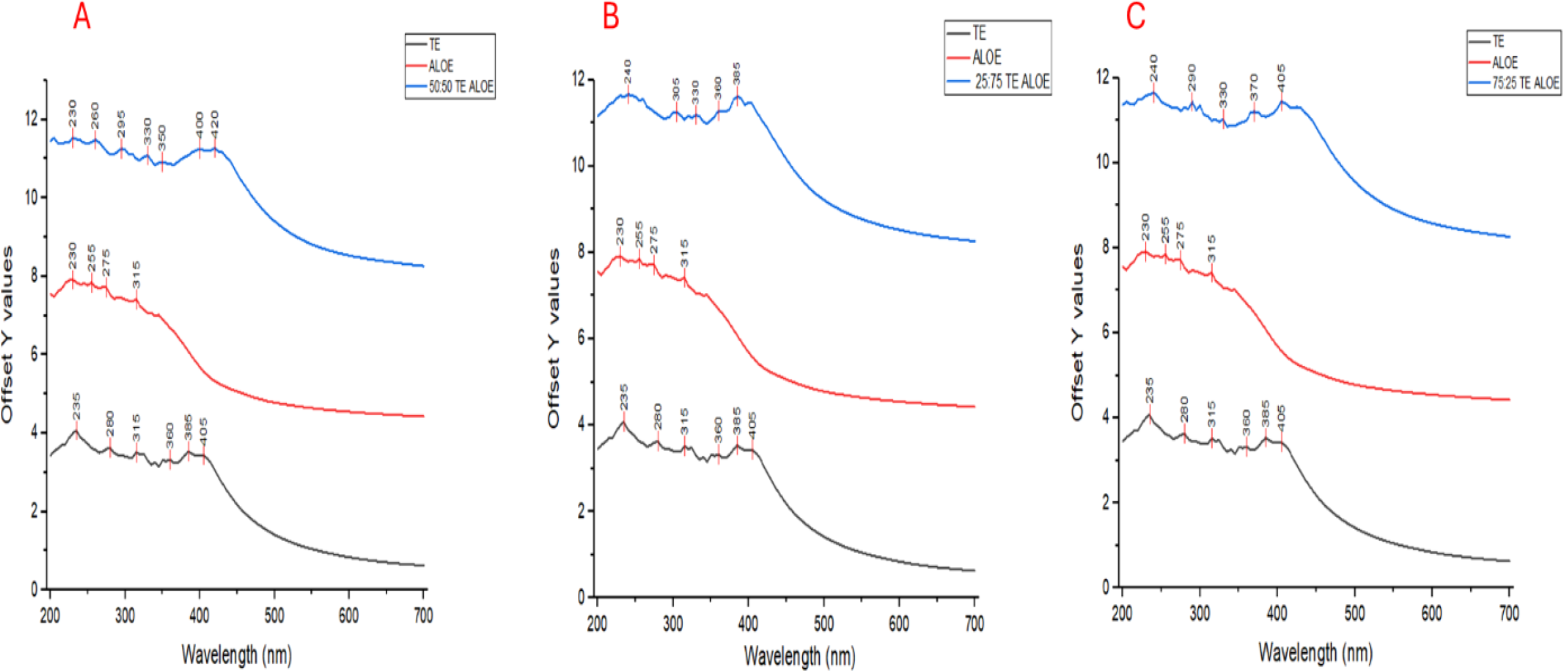
UV-Vis spectra of *T. elegans, Aloe vera*, and synthesized NPte at **(A)** 50:50, **(B)** 25:75, and **(C)** 75:25.

The UV-Vis spectra show distinct trends and shifts in absorption peaks across the three samples: *T. elegans* (TE) extract, *A. vera* (Aloe) extract, and the synthesized NPte formed using a 50:50 combination of *T. elegans* and *A. vera* (**Figure 6**). The *T. elegans* extract exhibits prominent peaks at approximately 235 nm, 280 nm, 315 nm, 360 nm, and 405 nm (**Figure 6**). The *A. vera* extract displays absorption peaks at 230 nm, 275 nm, 315 nm, 350 nm, 400 nm, and 420 nm. Compared to *T. elegans*, the peaks for *A. vera* are slightly shifted, with additional peaks at higher wavelengths (400 nm and 420 nm) (**Figure 6**). The 50:50 TE: Aloe nanoparticle sample shows a combination of features from both extracts, but with notable changes in peak intensity and wavelength shifts.

Peaks were observed at 230 nm, 260 nm, 295 nm, 330 nm, 400 nm, and 420 nm for treatments. Compared to the individual extracts, the nanoparticle spectrum indicated new peaks at 260 nm and 295 nm, indicating the formation of new molecular species or interactions between the phytochemicals of *T. elegans* and *A. vera* during nanoparticle synthesis (**Figure 6**). The increased intensity of the peaks at 400 nm and 420 nm reflects the successful stabilization of nanoparticles by *A. vera*.

The synthesized nanoparticles, using a 25:75 (TE: Aloe) ratio, show further shifts and the appearance of new peaks. Peaks are observed at 240 nm, 305 nm, 330 nm, 360 nm, and 385 nm. Compared to both *T. elegans* and *A. elegans*, the nanoparticle spectrum features a new peak at 240 nm and significant shifts in peaks previously seen at 315 nm (shifted to 305 nm) and 385 nm (enhanced intensity). The 75:25 (TE: Aloe) nanoparticle spectrum demonstrates significant modifications (**Figure 6**). The peaks are observed at 240 nm, 305 nm, 330 nm, 360 nm, and 385 nm. A new peak at 240 nm, alongside shifts in the existing peaks (from 315 nm to 330 nm and from 385 nm to 370 nm).

### 3.2 Bioassays

The Shapiro-Wilk normality test indicated that the variances of the tested nematode variable were not normally distributed (P ≤ 0.05); therefore, they were transformed accordingly. The interactions between time (24, 48, 72 hrs) and concentration (100:0, 25:75, 50:50, 75:25, and 0:100 (plant extract: *A. vera*) v/v) on all measured variables were not significant (P > 0.05).

#### 3.2.1 Effects of nanoparticles on second-stage juvenile (J2) hatch

Significant differences (P ≤ 0.05) were observed between synthesized nanoparticles and pure extract in relation to J2 hatch. Both the synthesized nanoparticles and the pure plant extracts exhibited an inhibitory effect, ranging from 37% to 74% on J2 hatch relative to the negative control treatment (**Table 1**). However, the J2 hatch inhibition was higher in eggs exposed to nanoparticles (54-74%) than those exposed to individual plant extracts (37-51%) (**Table 1**). The J2 hatch effects of the synthesized nanoparticles of the two plants did not differ at corresponding concentrations; however, differences were observed between different concentrations (**Table 1**). When nanoparticles were considered, the lowest juvenile hatch (6.9-7%) and highest hatch inhibitions (72-74%) were observed at 50:50 concentrations of plant extract to *A. vera*, whereas the highest J2 hatch (11-12%) and lowest hatch inhibition (54-57%) were observed at 75:25 (**Table 1**). An increase in the proportion of *A. vera* in the nanoparticle concentration increased the product’s J2 hatch inhibition (**Table 1**).

**Table 1:** Effects of nanoparticle concentrations on Meloidogyne incognita second-stage juvenile (J2) hatch.

Concentrations (Extract: *A. vera*)	Juvenile hatch	^y^Relative impact (%)
Negative control	^x^1. 3817^a^ (26.111)	^-^
*L. camara* (100 %)	1.1820^b^ (16.566)	-37
*A. vera* (100 %)	1.1424^bc^ (15.00)	-43
*T. elegans* (100 %)	1.0965^bcd^ (12.889)	-51
NPlc (75:25)	1.0523^cd^ (12.111)	-54
NPte (75:25)	1.0247^de^ (11.333)	-57
NPlc (25:75)	0.9363^ef^ (8.444)	-68
NPlc (25:75)	0.9025^f^ (9.222)	-65
NPlc (50:50)	0.7717^g^ (6.8889)	-74
NPte (50:50)	0.7707^g^ (7.222)	-72
F-value	19.34	
LSD_0.05_	0.1159	
P-Value	0.0000	

^x^Column means followed by the same letters are not significantly different at P ≤ 0.05 according to Fisher’s Least Significant Difference. Values in brackets are untransformed percentage J2 hatch means. ^y^Relative impact (%) = [(treatment/control) – 1] x 100.

Nanoparticles’ effect on *M. incognita* J2 hatch varied over the three exposure times (**Table 2**). The J2 hatch counts increased with the duration of exposure to the nanoparticles, with the highest J2 hatch observed after 72 hours (**Table 2**). The inhibitory effect of nanoparticles on J2 hatch was most pronounced within the first 24 hours of exposure, with the least inhibitory effects observed at 72 hours of exposure (**Table 2**).

**Table 2:** Effects of nanoparticles on Meloidogyne incognita second-stage juvenile (J2) hatch at different durations of exposure.

Time (hr)	J2 hatch	J2 hatch inhibition (%)
72	1.31^a^(21)	79^a^
48	1.09^b^(12)	88^b^
24	0.70^c^(5)	95^c^
F-Value	207.02	
LSD_0.05_	0.06	
P-Value	0.00	

Column means followed by the same letters are not significantly different at P ≤ 0.05 according to Fisher’s Least Significant Difference. Values in brackets are untransformed percentage J2 hatch means. J2 hatch inhibition (%) = (Total number of eggs−Number of J2 hatched) /Total number of eggs × 100.

#### 3.2.2 Effects of nanoparticles on second-stage juvenile mortality

Time and nanoparticles had statistically significant effects on J2 mortality (P ≤ 0.05). The effects of nanoparticles synthesized at varying concentrations on juvenile mortality revealed statistically significant (P ≤ 0.05) differences among the different concentrations. All synthesized nanoparticles and pure extracts resulted in significant juvenile mortality compared to the negative control, with nanoparticles generally performing better than the pure extracts (**Table 3**). The most pronounced *M. incognita* juvenile mortality was observed at the 50:50 and 25:75 extract-to-*A. vera* ratios for both plant extracts, at above 75% mortality, with no significant difference between these two concentration groups from both plants (**Table 3**). Relative to the negative control, juvenile mortalities of approximately 4 000% were observed after exposure to these concentration groups (**Table 3**). As with juvenile hatch, an increase in the proportion of *A. vera* in the nanoparticle concentration increased the product’s J2 mortality (**Table 3**).

**Table 3:** Effects of nanoparticles on *M. incognita* second-stage juvenile mortality at different synthesized concentrations.

Treatment (Extract: *A. vera*)	Juvenile mortality	^y^Relative impact (%)
NPlc (50:50)	1.9022^a^ (79.889)	4129
NPte (50:50)	1.9002^a^ (79.556)	4111
NPte (25:75)	1.8886^a^ (77.222)	3988
NPlc (25:75)	1.8766^ab^ (75.778)	3912
NPte (75:25)	1.8126^bc^ (65.111)	3347
NPlc (75:25)	1.7905^cd^ (62.000)	3182
*T. elegans* (100%)	1.7538^cd^ (57.444)	2941
*L. camara* (100%)	1.7321^d^ (54.889)	2806
*A. vera* (100%)	1.6385^e^ (43.889)	2223
Negative control	0.4147^f^ (1.889)	–
F-value	342.11	
LSD_0.05_	0.0653	
P-value	0.0000	

Column means followed by the same letters are not significantly different at P ≤ 0.05 according to Fisher’s Least Significant Difference. Values in brackets are untransformed percentage means of juvenile mortality. ^y^Relative impact (%) = [(treatment/control) – 1] x 100.

Juvenile mortality increased with the increase in duration of exposure to both the synthesized nanoparticles and pure plant extracts from 44-70% (**Table 4**). The highest percentage of juvenile mortality, at 70%, was observed after *M. incognita* J2 were exposed to nanoparticles for 72 h, whereas the least mortality, at 44%, was recorded after 24 h of exposure to nano-plant extracts (**Table 4**). The observed trend suggests that while cumulative mortality continued to rise with prolonged exposure, the actual amount by which J2 mortality increased gradually declined over time, indicating a potential reduction in the effectiveness of the treatments beyond the initial hours of exposure (**Table 4**).

**Table 4:** Effects of nanoparticles on juvenile mortality at different durations of exposure.

Time (hrs)	Juvenile mortality
72	1.7672^a^ (70.242)
48	1.6739^b^ (59.121)
24	1.5451^c^ (44.121)
F-value	85.28
LSD_0.05_	0.0341
P-value	0.0000

Column means followed by the same letters are not significantly different at P ≤ 0.05 according to Fisher’s Least Significant Difference. Values in brackets are untransformed juvenile mortality means.

## 4 Discussion

### 4.1 Nanoparticle synthesis

The present study was conducted to synthesize and characterize green nanoparticles (NPs) derived from *Lantana camara* (NPlc) and *Tabernaemontana elegans* (NPte) using *Aloe vera* (A. vera) as a reducing and stabilizing agent. Using TEM, ImageJ, UV-Vis spectroscopy, and SAED, the synthesized nanoparticles were characterized in terms of morphology, size distribution, optical properties, and crystallinity.

#### 4.1.1 Transmission electron microscopy

Transmission electron microscopy (TEM) provided visual confirmation of the morphological diversity of the nanoparticles, with structures ranging from cubic to triangular, plate-like, and irregular shapes. The size distribution revealed that the 50:50 extract-to-gel ratio resulted in the most uniformly dispersed particles with nanoscale dimensions, whereas more extract-heavy or gel-heavy formulations led to clustering and increased particle sizes.

Smaller nanoparticles (5–21 nm) are particularly relevant in the context of pest control, as studies have shown that particles in this range are more likely to interact with the nematode cuticle, penetrate tissues, and exert toxic effects (Khan et al., 2022). The aggregation observed at 75:25 and 25:75 ratios, forming clusters, aligns with studies by Mahawar et al. (2018), suggesting that insufficient or excessive stabilizing agents can disrupt the synthesis process, leading to reduced functionality. In this study, pure extracts produced particles outside the nanoscale range, which is likely due to aggregation resulting from the lack of stabilizing activity of the phytochemicals at certain ratios. Similar aggregation issues have been reported in the synthesis of green nanoparticles when the reducing and capping agents are not optimally balanced (Restrepo and Villa, 2021; Arcot et al., 2024). To mitigate aggregation and reduce particle size, optimization of synthesis conditions, such as adjusting the concentration of *A. vera* gel (as a natural stabilizer), applying sonication to disperse aggregates, and fine-tuning the solution pH to enhance electrostatic repulsion among nanoparticles, has been suggested (Pirsaheb et al., 2024). Future work should therefore investigate these optimization parameters to ensure stable nanoscale particle production suitable for biological applications.

Nanoparticle morphology is a critical determinant of their functional properties, influencing their behavior, efficacy, and potential applications in agriculture. These varied forms are indicative of the role plant extracts play in controlling nucleation and growth during the green synthesis process. Each morphology carries distinct advantages that impact its utility in pest management, nutrient delivery, and crop protection. Morphologies such as triangular and platelet-shaped nanoparticles exhibit high surface-area-to-volume ratios and sharp edge features, which significantly enhance contact with biological membranes and promote reactivity (Nagajyothi et al., 2020). For example, these morphologies can disrupt the structural integrity of nematodes or improve their adhesion to plant surfaces, thereby enhancing their efficiency as biopesticides (Kalaiselvi et al., 2017). Similarly, platelet-shaped nanoparticles offer a larger exposed surface area, facilitating better absorption or release of bioactive compounds when used for nutrient delivery or soil conditioning (Bushra et al., 2023). However, irregular shapes, while reactive, were associated with increased aggregation, which could hinder their dispersal in soil environments (Bushra et al., 2023). Therefore, maintaining shape control and preventing particle aggregation through stabilizing agents like *A. vera* polysaccharides is critical to ensure that the nanoparticles remain bioavailable and functional in agricultural settings.

#### 4.1.2 Selected area electron diffraction patterns

The structural properties of the synthesized nanoparticles were further analyzed using Selected Area Electron Diffraction (SAED). The diffraction patterns revealed concentric rings typical of polycrystalline materials, particularly in the 50:50 and 75:25 formulations. These rings correspond to the diffraction from multiple crystalline domains, indicating that the nanoparticles had well-defined, ordered atomic structures. However, it is important to clarify that SAED analysis provides information on crystallinity and lattice orientation, but it cannot be used to directly infer particle stability. Stability in nanoparticle systems is better evaluated through spectroscopic methods such as UV–Vis, where the persistence of absorption peaks without significant shifts or broadening over time indicates colloidal stability (Ray et al., 2015).

High crystallinity is a crucial factor in determining the function of nanoparticles. As reported by Zhang et al. (2020), polycrystalline nanoparticles exhibit improved catalytic activity and surface energy, which are advantageous in pest suppression scenarios. The 50:50 nanoparticles not only exhibited a smaller size and better dispersion but also the highest degree of crystallinity, suggesting that this formulation could offer an enhanced interaction with biological systems. In contrast, the more diffuse SAED patterns observed in the 25:75 blend indicated reduced order and higher levels of structural imperfection, which may affect stability and reactivity. Although the study did not test these particles for nematicidal activity, the high crystalline structure observed is a positive indicator, supporting the hypothesis that structural order could contribute to future bioactivity.

#### 4.1.3 Visual observation and UV-Vis spectra analysis

UV–Vis spectroscopy was used to characterize the optical properties of both the crude extracts and the synthesized nanoparticles. Unique absorption peaks observed in the visible region (especially around 400–420 nm) confirmed the formation of nanoparticles, while the presence of phenolic and flavonoid peaks in the UV region (215–300 nm) indicated the phytochemical profile of the plant extracts.

*Lantana camara* extracts showed peaks at 215, 240, and 300 nm, commonly associated with phenolic acids and flavonoids, while *A. vera* exhibited peaks at 230, 255, and 275 nm, reflecting the presence of flavonoids and polysaccharides (Dutta, 2023; Sayed-Ahmed and Shabana, 2024). These compounds are known to contribute both reducing power (phenolics) and colloidal stability (sugars), enabling the formation and maintenance of nanoparticles in solution. The absorbance at 255 nm and 275 nm is characteristic of flavonoids, which display absorption bands due to electronic transitions within their molecular structures (Kankala et al., 2020).

The UV-Vis spectra indicate that the nanoparticles exhibit unique absorbance characteristics not observed in the individual extracts, particularly in the UV-visible range. The broader and more intense peaks in the nanoparticle spectra, particularly in the 400–420 nm region, indicate the formation of surface plasmon resonance (SPR) peaks, a hallmark of nanoparticle formation. The peak shifts and overlap between the individual extracts in the 50:50 mixture reflect changes in the molecular structure and electronic properties of the phytochemicals upon nanoparticle synthesis.

These spectral changes suggest the potential involvement of polymerization processes, particularly the polymerization of phenolic compounds (Mizzi et al., 2020). Polyphenolic compounds, which are abundant in many plant extracts, are known to undergo oxidative polymerization, leading to the formation of nanoparticles (Guo et al., 2021). The presence of polysaccharides, such as those found in *A. vera*, likely plays a role in stabilizing these nanoparticles through interactions with the formed metal ions or other nanoparticles. The stabilization is likely facilitated by the hydrophilic nature of polysaccharides, which may form a protective shell around the nanoparticles, preventing aggregation and enhancing their stability in solution (Wang et al., 2022).

In the nanoparticle synthesis process, phenolic compounds likely undergo polymerization, while polysaccharides act as stabilizers (Shehzad et al., 2024). According to Bahari et al. (2023), the ratio of phenols to saccharides in the mixture is crucial to the nanoparticle formation process, as it determines both the reduction and stabilization mechanisms. The observed shifts in the UV-Vis spectra, particularly the changes in the absorbance intensity and the appearance of new peaks, suggest a balance between these two classes of compounds. The higher concentration of *A. vera* (75%) in the 25:75 (LC: Aloe) mixture leads to the formation of a more stable nanoparticle system, as evidenced by the increased intensity of the peaks at 400–420 nm and the shifts in the UV region. While the exact ratio of phenols to polysaccharides cannot be directly determined from the UV-Vis spectra, the presence of both classes of compounds in the extracts is confirmed. Phenolic compounds are likely responsible for the redox reactions required for nanoparticle reduction, while polysaccharides provide the necessary stabilization and prevent aggregation of nanoparticles (Sahraeian et al., 2024).

Importantly, the appearance of new peaks and the broadening of absorption bands in the nanoparticle mixtures suggest molecular interactions and potential polymerization between phenolic compounds and polysaccharides (Mizzi et al., 2020; Guo et al., 2021). These chemical interactions help explain the stability and dispersity observed in the nanoparticles, further confirming the functional role of the plant-derived biomolecules.

#### 4.1.4 Possible mode of nanoparticle synthesis

The formation of the nanoparticles is likely driven by synergistic redox and stabilization reactions involving plant-derived phytochemicals. Reducing sugars, such as the polysaccharides in *A. vera*, serve as natural electron donors, initiating the reduction of phytochemicals into nanoparticle nuclei (Zhang et al., 2020; Pirsaheb et al., 2024). In the absence of external chemicals or metals, reducing sugars serve as natural electron donors, driving the reduction process necessary for nanoparticle formation (Mizzi et al., 2020). Their role is pivotal because they initiate and sustain the conversion of bioactive compounds in plant extracts into nanoscale particles through a series of biochemical reactions. These sugars contain functional groups, such as aldehydes and ketones, which are highly reactive and capable of transferring electrons to the precursor molecules, facilitating the reduction of reactive species into stable nanoparticles (Bahari et al., 2023). This electron transfer is central to the nucleation phase, where the initial formation of nanoparticles occurs (Tapia-Hernández et al., 2018).

Phenolic and flavonoid compounds from both *T. elegans* and *L. camara* further enhance this process by facilitating electron transfer and stabilizing the particles through hydrogen bonding and electrostatic interactions. Once nucleation occurs, the nanoparticles grow and stabilize via the adsorption of polysaccharides and other biomolecules onto their surface, preventing agglomeration and maintaining a uniform size (Mittal et al., 2013). The ratio of reducing and stabilizing agents in the mixture appears to be a key factor in determining the final nanoparticle morphology and performance. The stabilization effect is crucial in maintaining the functional properties of the nanoparticles, such as their size, shape, and bioactivity (Sharma et al., 2020). For example, in the synthesis of nanoparticles using *A. vera* gel as a reducing agent, the polysaccharides in the gel stabilize the nanoparticles by interacting with their surface through hydrogen bonding and van der Waals forces (Mittal et al., 2013). This interaction not only prevents particle aggregation but also enhances the dispersity of the nanoparticles, making them more effective for applications like nematode management (Yadav and Yadav, 2018). Higher concentrations of carbohydrates often favor rapid nucleation, resulting in smaller and more uniform nanoparticles. Conversely, slower nucleation and sustained growth can result in larger nanoparticles with diverse morphologies (Jadoun et al., 2021). Verma et al. (2020) explained that these controlled mechanisms are critical when plant extracts are used as the only source for nanoparticle synthesis, as the absence of chemical stabilizers or metal ions makes the role of bioactive compounds, including carbohydrates, even more significant.

The possible mode of action of carbohydrates in the nanoparticle synthesis process is rooted in their ability to act as natural reductants and stabilizers. According to Ovais et al. (2018), during the synthesis, the hydroxyl groups in carbohydrates interact with the bioactive compounds within the plant extract, reducing them into reactive intermediates that subsequently form nanoparticles. This reaction is catalyzed by the inherent redox potential of carbohydrates, which allows them to donate electrons without requiring external catalysts. Moreover, the protective carbohydrate layer formed on the nanoparticles surface enhances their colloidal stability, making them suitable for long-term storage and various applications.

From the current study, nanoparticles synthesized from *T. elegans* and *L. camara*, the reducing sugars and other phytochemicals work in concert to produce stable nanoparticles capable of targeting nematodes effectively. These nanoparticles likely exhibit enhanced bioactivity due to their nanoscale properties, which will improve interaction with nematodes and reduce the overall amount of material needed for pest management, making the approach both cost-effective and sustainable (Mittal et al., 2013). The nanoparticle’s extremely small size enables it to attach to and disrupt the nematode’s cuticle, causing structural damage, dehydration, and ultimately leading to death. At the same time, these nanoparticles can generate reactive oxygen species, which induce oxidative stress, leading to DNA damage, protein denaturation, and interference with essential metabolic processes. Once inside the nematode, they may also disrupt neurotransmission, enzyme activity, and reproductive functions, thereby reducing mobility, feeding, and egg hatching. The phytochemicals from the plant extract that remain on the nanoparticle surface can contribute additional nematicidal effects by acting as natural toxins, further weakening or killing the nematode. From the current study, a balanced 50:50 ratio likely provides sufficient reducing power without overwhelming the stabilizing system, producing small, uniform, and highly crystalline nanoparticles.

### 4.2 Bioassays test

Nanotechnology provides a sustainable approach to pest control by enhancing the efficacy of plant-based bioactive compounds. In this study, nanoparticles synthesized from *L. camara* and *T. elegans* significantly inhibited the hatch and increased the mortality of *Meloidogyne incognita* second-stage juveniles (J2) under *in vitro* conditions.

The influence of exposure duration and nanoparticle concentrations revealed significant trends and variations in nematode control efficacy. The inhibition of J2 hatch was time-dependent, with the strongest suppression observed within the first 24 hours, followed by gradual increases in hatch rate over 48 and 72 hours. This pattern aligns with previous findings by Nazir et al. (2019) and Nagachandrabose et al. (2024), who reported time-dependent hatch inhibition and nematode mortality following exposure to nanoparticles. Variations in hatch over time may reflect differences in egg developmental stages or adaptive physiological responses among surviving embryos (Mahawar et al., 2018; Khan et al., 2022).

Nazir et al. (2019) investigated the *in vitro* effectiveness of silver nanoparticles (AgNPs) against *M. incognita* and observed a direct relationship between nanoparticle concentration and the mortality of J2. It was observed that higher concentrations of AgNPs resulted in increased mortality rates of J2, with significant inhibition of J2 hatch observed over time. The inhibition was directly proportional to the duration of exposure, increasing over a six-day period. Their results indicated that all tested nanoparticles significantly reduced J2 hatch percentages and increased J2 mortality compared to controls. However, Khosa et al. (2020) demonstrated that prolonged exposure of *M. incognita* J2 to plant extracts often correlates with increased physiological responses in nematodes due to gradual activation of internal biochemical mechanisms. Longer exposure times may enhance metabolic activity and physiological processes within the eggs, enabling more efficient development and J2 hatching (Danish et al. 2025).

According to Ebrahimi (2015), observations on the J2 hatch of *Globodera pallida*, a cyst nematode, increased progressively with time when exposed to favorable conditions, suggesting that duration is a critical factor in the successful emergence of J2. Furthermore, Jensen (2018) found that the hatch of *Heterodera glycines* J2 was significantly higher after 72 h compared to shorter exposure durations, likely due to the extended time allowing for the accumulation of metabolic precursors required for hatching.

Conversely, some studies have reported deviations from this trend, particularly under adverse environmental or chemical conditions. For instance, Ali et al. (2021) observed that prolonged exposure to certain nematicidal agents could inhibit J2 hatch in *M. incognita*, regardless of duration. The researchers attributed this to the toxic effects of the compounds, which disrupted embryonic development and reduced J2 hatchability over time. Similarly, Santos (2017) reported that extreme environmental factors, such as high temperatures or saline conditions, can delay or suppress the hatch of *Meloidogyne* (*M. incognita*) nematode juveniles, even with extended durations, suggesting that hatch trends can vary based on external stressors. The increase in J2 hatch observed in the current study underlines the importance of optimizing exposure durations to achieve desired outcomes in nematode management. While extended durations may favor J2 hatch under untreated or mild conditions, incorporating inhibitory agents such as nanoparticles could alter these dynamics.

Nanoparticle concentrations significantly influenced the inhibition of *Meloidogyne* juvenile hatch, as evident in the differences between untreated controls, raw plant extracts, and nanoparticle treatments. The negative control exhibited the highest J2 hatching rates, reflecting the natural hatch potential of J2 from eggs in the absence of a nanoparticle. Interestingly, treatments representing 100% *A. vera*, *L. camara*, and *T. elegans* extracts did not produce J2 hatching rates that differed significantly from the untreated control, suggesting moderate nematicidal activity of the raw extracts. These findings are consistent with previous studies by Abbassy et al. (2017) and Rajeshkumar et al. (2020), who emphasized that nanoparticles exhibit enhanced bioactivity compared to bulk plant extracts due to their unique physicochemical properties.

Nanoparticles synthesized from *L. camara* and *T. elegans* at 50:50 and 25:75 ratios with *A. vera* demonstrated significant inhibition of juvenile hatch, underscoring their superior nematicidal efficacy. This enhanced activity can be attributed to the high surface area-to-volume ratio of nanoparticles, which improves their interaction with biological membranes, facilitating better delivery of bioactive compounds. Rajeshkumar et al. (2020) highlighted that nanoparticles derived from plant extracts showed greater bioactivity against *Meloidogyne* spp, particularly due to their ability to effectively penetrate nematode eggs and disrupt internal metabolic processes. Plant-nematode interactions involve various compounds that function as repellents, attractants, hatch stimulants or inhibitors, and nematotoxicants, either naturally occurring or induced in response to the presence of nematodes (Sharma et al., 2023).

Abbassy et al. (2017) reported that nanoparticles synthesized from horseweed (*Conyza dioscoridis* L.) extracts and metals effectively suppressed the hatch and mobility of *M. incognita* J2, demonstrating the potential of nanoscale formulations in enhancing nematicidal activity. Similarly, Nassar (2016) found that nanoparticles derived from Annual nettle (*Urtica urens* L.) increased nematicidal efficacy elevenfold compared to raw plant extracts. These studies collectively reinforce the conclusion that nanoparticle synthesis amplifies the bioactivity of plant-derived compounds against plant-parasitic nematodes.

Conversely, some studies suggest that the efficacy of nanoparticles may vary depending on factors such as environmental conditions, nanoparticle stability, and the specific plant-parasitic nematode species targeted. For instance, Castillo-Henríquez et al. (2020) raised concerns regarding the variability in nanoparticle effects under different soil compositions and moisture levels, which may influence their bioavailability. Additionally, studies by Wahab et al. (2024) noted that while nanoparticles generally exhibit superior activity, their prolonged stability and potential environmental persistence warrant careful consideration in agricultural applications.

Exposure duration played a critical role in influencing J2 mortality rates in *Meloidogyne* nematodes. Mortality significantly increased with extended exposure, with the highest rates recorded at 72 h and the lowest at 24 h. This time-dependent efficacy highlights the progressive impact of nanoparticles on *M. incognita* J2, likely due to prolonged interaction and disruption of nematode physiology over time. These findings are consistent with Nassar (2016), who reported similar time-dependent nematicidal effects in nanoparticle formulations derived from *Azadirachta indica* (Neem). The gradual release of bioactive compounds over time was noted to enhance mortality in *M. incognita*. However, Bordoloi (2019) observed that a 100% concentration of *L. camara* leaf extract was highly effective at 48 h, suggesting that specific plant extracts may exhibit rapid efficacy under certain conditions. This discrepancy emphasizes the importance of optimizing exposure duration based on the source and formulation of the treatment.

Nanoparticle concentration further influenced J2 mortality. An increase in the proportion of *A. vera* in the nanoparticle concentration increased the product’s J2 mortality, with nanoparticles synthesized at 50:50 and 25:75 ratios (extract: *A. vera*) exhibiting the highest mortality rates, significantly outperforming the pure plant extracts and the negative control. This suggests a synergistic interaction between *A. vera* and the plant extracts in the nanoparticle formulations. *Aloe vera* bioactive compounds, such as polysaccharides and anthraquinones, likely contributed to this enhanced efficacy by stabilizing and reducing the nanoparticles during synthesis. Prasad et al. (2021) made similar observations, noting that *A. vera*’s bioactive properties enhance the nematicidal effects of nanoparticles by improving stability and bioavailability. This is further supported by Khan et al. (2023), who demonstrated that nanoparticles enhance the delivery of active compounds, increasing their interaction with nematodes and disrupting their biological processes more effectively.

The superior performance of synthesized nanoparticles compared to raw plant extracts underscores the advantages of nanotechnology in nematode management. Nanoparticles, with their high surface area-to-volume ratio and enhanced bioactivity, provide a more efficient delivery system for bioactive compounds. This property ensures that the active ingredients penetrate nematode cuticles and disrupt their physiological functions more effectively. Studies on green-synthesized nanoparticles derived from plants such as *Conyza dioscoridis* and *Urtica urens* have similarly demonstrated significant improvements in nematicidal activity compared to their bulk counterparts (Abbassy et al., 2017).

Interestingly, the proportional increase in *A. vera* in nanoparticle synthesis amplified their nematicidal activity, indicating its critical role as a reducing and stabilizing agent. This aligns with findings by Castillo-Henríquez et al. (2020), who noted that plant-based nanoparticles exhibit increased efficacy due to the synergistic actions of their bioactive constituents. The addition of *A. vera* likely enhances the nanoparticles structural integrity, chemical composition and delivery efficiency, optimizing their nematicidal potential.

While the results align with many studies supporting the efficacy of nanoparticles in pest management, some research raises concerns about variability in performance due to environmental factors or differences in nematode species. For instance, Dang et al. (2018) noted that the effectiveness of nanoparticles could vary depending on soil composition and moisture levels, which influence bioavailability. Such variability underscores the need for field-based studies to validate the consistency of laboratory findings under real-world conditions.

An ideal, green-synthesized nanoparticle should possess a uniform structure, strong absorption properties, and enhanced stability. Uniformity in particle size and shape is essential, as it directly influences the physical, optical, and catalytic properties of the nanoparticles. Consistent morphology ensures predictable interactions with biological systems and reliable performance in various applications. Strong absorbance features, usually reflected in UV–Vis spectra, indicate efficient surface plasmon resonance and effective reduction of metal ions during synthesis, key indicators of successful nanoparticle formation. Enhanced stability, on the other hand, prevents aggregation and degradation over time, maintaining the nanoparticles’ functionality and biocompatibility. Achieving these characteristics through green synthesis methods, which utilize plant extracts or other environmentally friendly reducing agents, not only minimizes the use of toxic chemicals but also promotes sustainability and safer nanomaterial production.

## 5 Conclusion

Green nanoparticles with potent nematicidal activity against *Meloidogyne incognita* were successfully synthesized. The efficacy of the nanoparticles was influenced by concentration and exposure duration, with 50:50 and 25:75 extract-to-gel ratios being the most effective. The high surface reactivity and synergistic combination of phytochemicals and *A. vera* components contributed to superior inhibition of juvenile hatch and mortality. These findings underscore the potential of plant-based nanoparticles as sustainable alternatives to synthetic nematicides. Further research should focus on field validation, environmental safety assessment, and mechanistic studies to optimize the application of these methods in integrated nematode management programs.

## Data Availability

The original contributions presented in the study are included in the article/supplementary material. Further inquiries can be directed to the corresponding author.

## References

[B1] Abbassy M. A. Abdel-Rasoul M. A. Nassar A. M. Soliman B. S. (2017). Nematicidal activity of silver nanoparticles of botanical products against root-knot nematode, Meloidogyne incognita. Arch. Phytopathol. Plant Prot. 50, 910–920. doi: 10.1080/03235408.2017.1405608, PMID: 41799851

[B2] Abd-Elgawad M. M. (2024a). Upgrading strategies for managing nematode pests on profitable crops. Plants 13, 2–7. doi: 10.3390/plants13111558, PMID: 38891366 PMC11174438

[B3] Abd-Elgawad M. M. (2024b). “ Revolutionizing nematode management–nanomaterials as a promising approach for managing economically important plant-parasitic nematodes: Current Knowledge and Future Challenges,” in Nanotechnology and plant disease management. Eds. Mahmood I. Ansari R. A. Rizvi R. ( Taylor and Francis, Boca Raton), 23–44.

[B4] Ali S. S. Ahmed F. H. Khan Z. R. Rahman A. B. (2021). Influence of environmental factors on nanoparticle efficacy in nematode management. Agric. J. 76, 254–263.

[B5] Arcot Y. Iepure M. Hao L. Min Y. Behmer S. T. Akbulut M. (2024). Interactions of foliar nanopesticides with insect cuticle facilitated through plant cuticle: effects of surface chemistry and roughness-topography-texture. Plant Nano Biol. 7, 2–6. doi: 10.1016/j.plana.2024.100062, PMID: 41842036

[B6] Bahari N. Hashim N. Abdan K. Md Akim A. Maringgal B. Al-Shdifat L. (2023). Role of honey as a bifunctional reducing and capping/stabilizing agent: application for silver and zinc oxide nanoparticles. Nanomaterials 13, 3–7. doi: 10.3390/nano13071244, PMID: 37049336 PMC10097146

[B7] Bordoloi K. (2019). Mechanism of Lantana camara leaf extracts in the management of Meloidogyne incognita on tomato. Assam Agricultural University, India.

[B8] Bushra R. Ahmad M. Seidi F. Song J. Jin Y. Xiao H. (2023). Polysaccharide-based nanoassemblies: From synthesis methodologies and industrial applications to future prospects. Adv. Colloid Interface Sci. 318, 3–26. doi: 10.1016/j.cis.2023.102953, PMID: 37399637

[B9] Canaparo R. Foglietta F. Limongi T. Serpe L. (2020). Biomedical applications of reactive oxygen species generation by metal nanoparticles. Materials 14, 53. doi: 10.3390/ma14010053, PMID: 33374476 PMC7795539

[B10] Castillo-Henríquez L. Alfaro-Aguilar K. Ugalde-Álvarez J. Vega-Fernández L. Montes de Oca-Vásquez G. Vega-Baudrit J. R. (2020). Green synthesis of gold and silver nanoparticles from plant extracts and their possible applications as antimicrobial agents in the agricultural area. Nanomaterials 10, 2–10. doi: 10.3390/nano10091763, PMID: 32906575 PMC7558319

[B11] Dang F. Jiang Y. Li M. Zhong H. Peijnenburg W. G. Shi W. . (2018). Oral bioaccessibility of silver nanoparticles and ions in natural soils: Importance of soil properties. Environ. pollut. 243, 364–373. doi: 10.1016/j.envpol.2018.08.092, PMID: 30199811

[B12] Danish M. Altaf M. Shahid M. Robab M. I. Amir M. Ahmed S. M. . (2025). Synergistic interaction of silicon dioxide nanoparticles (SiO2NPs) and Pseudomonas fluorescens to combat Meloidogyne incognita infestation: enhancing growth, biochemical, and antioxidant activities in Trachyspermum ammi (L.). J. Soil Sci. Plant Nutr. 25, 1505–1523. doi: 10.1007/s42729-025-02219-z, PMID: 41841152

[B13] Dharajiya D. Pagi N. Jasani H. Patel P. (2017). Antimicrobial activity and phytochemical screening of Aloe vera (Aloe barbadensis Miller). Int. J. Curr. Microbiol. Appl. Sci. 6, 2–6. doi: 10.20546/ijcmas.2017.603.246

[B14] Dutta H. (2023). “ Potential of polysaccharide nanoparticles in foods,” in Nanotechnology horizons in food process engineering. Eds. Megh R. G. Junaid A. M. Satish K. Ritesh B. W. ( Academic Press, New York), 166–248.

[B15] Ebrahimi N. (2015). Quantifying live potato cyst nematodes (Globodera rostochiensis and G. pallida) and cultural practices to reduce their survival. Belgium: Ghent University. 10.20546/ijcmas.2017.603.246

[B16] Gautam K. Singh H. Sinha A. K. (2025). Nanotechnology in plant nanobionics: mechanisms, applications, and future perspectives. Adv. Biol. 9, 3–6. doi: 10.1002/adbi.202400589, PMID: 39936866

[B17] Guo H. Han F. Shang H. Xiong S. Huynh M. Thistle L. . (2021). New insight into naturally formed nanosilver particles: Role of plant root exudates. Environ. Sci. Nano 8, 1580–1592. doi: 10.1039/D0EN01188F, PMID: 41842999

[B18] Hasan M. Ahmad F. Malan P. Nadeem H. Asif M. Khan A. . (2021). Use of weed plants against Meloidogyne incognita in spinach involves reduction of gall disease from roots. Acta Agriculturae Scandinavica Section B—Soil Plant Sci. 71, 498–506. doi: 10.1080/09064710.2021.1924250, PMID: 41799851

[B19] Horie M. Tabei Y. (2021). Role of oxidative stress in nanoparticles toxicity. Free Radical Res. 55, 331–342. doi: 10.1080/10715762.2020.1859108, PMID: 33336617

[B20] Jadoun S. Arif R. Jangid N. K. Meena R. K. (2021). Green synthesis of nanoparticles using plant extracts: A review. Environ. Chem. Lett. 19, 355–374. doi: 10.1007/s10311-020-01074-x, PMID: 41841152

[B21] Jensen J. P. (2018). Effects of nematicidal seed treatments on the biology of the soybean cyst nematode, Heterodera glycines. Iowa State University, United States.

[B22] Kalaiselvi D. Sundararaj P. Premasudha P. Hafez S. L. (2017). Nematicidal activity of green synthesized silver nanoparticles using plant extracts against root-knot nematode Meloidogyne incognita. Int. J. Nematol. 27, 81–94.

[B23] Kankala R. K. Han Y. H. Na J. Lee C. H. Sun Z. Wang S. B. . (2020). Nanoarchitectured structure and surface biofunctionality of mesoporous silica nanoparticles. Adv. Mater. 32, 2–12. doi: 10.1002/adma.201907035, PMID: 32319133

[B24] Khan A. Bani Mfarrej M. F. Danish M. Shariq M. Khan M. F. Ansari M. S. . (2022). Synthesized copper oxide nanoparticles via the green route act as antagonists to pathogenic root-knot nematode, Meloidogyne incognita. Green Chem. Lett. Rev. 15, 491–507. doi: 10.1080/17518253.2022.2096416, PMID: 41799851

[B25] Khan M. R. Haroun S. A. Rizvi T. F. (2023). “ Novel nanomaterials and nanoformulations for nematode management in agricultural crops,” in Novel biological and biotechnological applications in plant nematode management. Ed. Gopala R. K. ( Springer Nature, Singapore), 227–243.

[B26] Khosa M. C. Dube Z. P. Tselanyane M. Fouche G. De Waele D. Daneel M. S. (2020). Density-dependent growth response and sensitivity of Meloidogyne incognita to Maerua angolensis and Tabernaemontana elegans: Card model. Acta Agriculturae Scandinavica Section B—Soil Plant Sci. 70, 24–30. doi: 10.1080/09064710.2019.1663913, PMID: 41799851

[B27] Kumar V. Khan M. R. Walia R. K. (2020). Crop loss estimations due to plant-parasitic nematodes in major crops in India. Natl. Acad. Sci. Lett. 43, 409–412. doi: 10.1007/s40009-020-00895-2, PMID: 41841152

[B28] Mahawar H. Prasanna R. Singh S. B. Nain L. (2018). Influence of silver, zinc oxide and copper oxide nanoparticles on the cyanobacterium Calothrix elenkinii. BioNanoScience 8, 802–810. doi: 10.1007/s12668-018-0543-2, PMID: 41841152

[B29] Malahlela M. Thibane V. S. Mudau F. N. (2021). Nematocidal activity of fermented extracts from Lantana camara plant parts against Meloidogyne javanica on tomato. Int. J. Veg. Sci. 27, 20–28. doi: 10.1080/19315260.2019.1697981, PMID: 41799851

[B30] Manimegalai T. Selvam S. Devi L. A. Vinothini A. (2020). Green synthesized zinc oxide nanoparticles from Tagetes erecta aqueous root extract against Meloidogyne incognita. Biochem. Cell. Arch. 24, 3–8. doi: 10.51470/bca.2024.24.1.149

[B31] Mikušová V. Mikuš P. (2021). Advances in chitosan-based nanoparticles for drug delivery. Int. J. Mol. Sci. 22, 2–15. doi: 10.3390/ijms22179652, PMID: 34502560 PMC8431817

[B32] Mittal A. K. Chisti Y. Banerjee U. C. (2013). Synthesis of metallic nanoparticles using plant extracts. Biotechnol. Adv. 31, 346–356. doi: 10.1016/j.biotechadv.2013.01.003, PMID: 23318667

[B33] Mizzi L. Chatzitzika C. Gatt R. Valdramidis V. (2020). HPLC analysis of phenolic compounds and flavonoids with overlapping peaks. Food. Technol. Biotechnol. 58, 12–19. doi: 10.17113/ftb.58.01.20.6395, PMID: 32684783 PMC7365340

[B34] Nagachandrabose S. Sivathanu L. Pon S. M. Kalimuthu R. Somasundaram P. Subramanian K. S. (2024). Nano-emulsion formulation of nematode egg parasitic fungus, Pochonia chlamydosporia to control Meloidogyne incognita infecting tomato. Biocontrol Sci. Technol. 34, 1–17. doi: 10.1080/09583157.2023.2294216, PMID: 41799851

[B35] Nagajyothi P. C. Prabhakar Vattikuti S. V. Devarayapalli K. C. Yoo K. Shim J. Sreekanth T. V. M. (2020). Green synthesis: photocatalytic degradation of textile dyes using metal and metal oxide nanoparticles-latest trends and advancements. Crit. Rev. Environ. Sci. Technol. 50, 2–5. doi: 10.1080/10643389.2019.1705103, PMID: 41799851

[B36] Nasr A. Yousef A. F. Hegazy M. G. Abdel-Mageed M. A. Elshazly E. H. Gad M. . (2025). Biosynthesized silver nanoparticles mitigate charcoal rot and root-knot nematode disease complex in faba bean. Physiol. Mol. Plant Pathol. 136, 102610. doi: 10.1016/j.pmpp.2025.102610, PMID: 41842036

[B37] Nassar A. M. (2016). Research article effectiveness of silver nanoparticles of extracts of Urtica urens (Urticaceae) against root-knot nematode Meloidogyne incognita. Asian J. Nematol. 5, 14–19. doi: 10.3923/ajn.2016.14.19

[B38] Nazir K. Mukhtar T. Javed H. (2019). *In Vitro* Effectiveness of silver nanoparticles against root-knot nematode (Meloidogyne incognita). Pak. J. Zool. 51, 2–7. doi: 10.17582/journal.pjz/2019.51.6.2077.2083

[B39] Ovais M. Khalil A. T. Islam N. U. Ahmad I. Ayaz M. Saravanan M. . (2018). Role of plant phytochemicals and microbial enzymes in biosynthesis of metallic nanoparticles. Appl. Microbiol. Biotechnol. 102, 2–6. doi: 10.1007/s00253-018-9146-7, PMID: 29882162

[B40] Pirsaheb M. Gholami T. Seifi H. Dawi E. A. Said E. A. Hamoody A. H. M. . (2024). Green synthesis of nanomaterials by using plant extracts as reducing and capping agents. Environ. Sci. pollut. Res. 31, 2–8. doi: 10.1007/s11356-024-32983-x, PMID: 38523214

[B41] Prasad A. R. Williams L. Garvasis J. Shamsheera K. O. Basheer S. M. Kuruvilla M. . (2021). Applications of phytogenic ZnO nanoparticles: A review on recent advancements. J. Mol. Liq. 331, 1–7. doi: 10.1016/j.molliq.2021.115805, PMID: 41842036

[B42] Punniyakotti P. Vinayagam S. Rajamohan R. Priya S. D. Moovendhan M. Sundaram T. (2024). Environmental fate and ecotoxicological behaviour of pesticides and insecticides in non-target environments: Nanotechnology-based mitigation strategies. J. Environ. Chem. Eng. 12, 3–9. doi: 10.1016/j.jece.2024.113349, PMID: 41842036

[B43] Rajeshkumar S. Venkat Kumar S. Ramaiah A. Banu M. (2020). Application of nanoparticles in nematode control: A review. Nanotechnology Agric. 3, 67–80.

[B44] Ray T. R. Lettiere B. de Rutte J. Pennathur S. (2015). Quantitative characterization of the colloidal stability of metallic nanoparticles using UV–Vis absorbance spectroscopy. Langmuir 31, 3–6. doi: 10.1021/la504511j, PMID: 25730093

[B45] Restrepo C. V. Villa C. C. (2021). Synthesis of silver nanoparticles, influence of capping agents, and dependence on size and shape: A review. Environ. Nanotechnology Monit. Manage. 15, 2–9. doi: 10.1016/j.enmm.2021.100428, PMID: 41842036

[B46] Rocha T. L. Polez V. L. P. de Souza Viol L. C. Pimentel R. R. Biscaia D. Pinheiro J. B. (2022). “ Use of natural and residual resources for the sustainable management of phytonematodes: challenges and future trends,” in Sustainable management of nematodes in agriculture. Eds. Chaudhary K. K. Meghvansi M. K. ( Springer, Cham), 3–28.

[B47] Sahraeian S. Rashidinejad A. Golmakani M. T. (2024). Recent advances in the conjugation approaches for enhancing the bioavailability of polyphenols. Food Hydrocolloids 146, 3–9. doi: 10.1016/j.foodhyd.2023.109221, PMID: 41842036

[B48] Santos F. M. (2017). Environmentally sustainable solutions for Root-knot nematodes control (Portugal: Universidade de Coimbra).

[B49] Sayed-Ahmed K. Shabana Y. M. (2024). “ The physiological impacts of nanoparticle size, morphology, and concentration on the phytopathogens causing plant biotic stress,” in Nanoparticles in plant biotic stress management. Eds. Khan M. Chen J. T. ( Springer Nature, Singapore), 2–11.

[B50] Sharma N. Khanna K. Jasrotia S. Kumar D. Bhardwaj R. Ohri P. (2023). Metabolites and chemical agents in the plant roots: an overview of their use in plant-parasitic nematode management. Nematology 25, 243–257. doi: 10.1163/15685411-bja10220, PMID: 40696532

[B51] Sharma B. Singh I. Bajar S. Gupta S. Gautam H. Kumar P. (2020). Biogenic silver nanoparticles: evaluation of their biological and catalytic potential. Indian J. Microbiol. 60, 468–474. doi: 10.1007/s12088-020-00889-0, PMID: 33087996 PMC7539258

[B52] Shehzad Q. Liu Z. Zuo M. Wang J. (2024). The role of polysaccharides in improving the functionality of zein coated nanocarriers: Implications for colloidal stability under environmental stresses. Food Chem. 431, 2–13. doi: 10.1016/j.foodchem.2023.136967, PMID: 37604006

[B53] Shriniwas P. P. Subhash T. K. (2017). Antioxidant, antibacterial and cytotoxic potential of silver nanoparticles synthesized using terpenes rich extract of Lantana camara L. leaves. Biochem. Biophys. Rep. 10, 76–81. 29114571 10.1016/j.bbrep.2017.03.002 PMC5637243

[B54] Singh H. Desimone M. F. Pandya S. Jasani S. George N. Adnan M. . (2023). Revisiting the green synthesis of nanoparticles: uncovering influences of plant extracts as reducing agents for enhanced synthesis efficiency and its biomedical applications. Int. J. Nanomed. 18, 4727–4750. doi: 10.2147/IJN.S419369, PMID: 37621852 PMC10444627

[B55] Smalley V. K. (1988). Colonization and parasitism of females and eggs of Meloidogyne incognita by fungi. University of Tennessee, Knoxville.

[B56] Sudhimon S. Kumar M. M. Yamini S. Devi T. A. Sumathi S. Sudagar J. (2024). Bio prospecting of Aloe barbadensis miller (Aloe vera) for silver nanoparticles against breast cancer: A review. J. King Saud Univ. Sci. 36, 3–9. doi: 10.1016/j.jksus.2024.103317, PMID: 41842036

[B57] Tapia-Hernández J. A. Rodríguez-Felix F. Juárez-Onofre J. E. Ruiz-Cruz S. Robles-García M. A. Borboa-Flores J. . (2018). Zein-polysaccharide nanoparticles as matrices for antioxidant compounds: A strategy for prevention of chronic degenerative diseases. Food Res. Int. 111, 451–471. doi: 10.1016/j.foodres.2018.05.036, PMID: 30007708

[B58] Uddin M. N. Roy S. C. Mamun A. A. Mitra K. Haque M. Z. Hossain M. L. (2020). Phytochemicals and in-vitro antioxidant activities of Aloe vera gel. J. Bangladesh Acad. Sci. 44, 33–41. doi: 10.3329/jbas.v44i1.48561

[B59] Verma M. L. Dhanya B. S. Rani V. Thakur M. Jeslin J. Kushwaha R. (2020). Carbohydrate and protein based biopolymeric nanoparticles: current status and biotechnological applications. Int. J. Biol. Macromol. 154, 390–412. doi: 10.1016/j.ijbiomac.2020.03.105, PMID: 32194126

[B60] Wahab A. Muhammad M. Ullah S. Abdi G. Shah G. M. Zaman W. . (2024). Agriculture and environmental management through nanotechnology: Eco-friendly nanomaterial synthesis for soil-plant systems, food safety, and sustainability. Sci. Total Environ. 926, 2–9. doi: 10.1016/j.scitotenv.2024.171862, PMID: 38527538

[B61] Wang X. Fan Y. Yan J. Yang M. (2022). Engineering polyphenol-based polymeric nanoparticles for drug delivery and bioimaging. Chem. Eng. J. 439, 3–7. doi: 10.1016/j.cej.2022.135661, PMID: 41842036

[B62] Yadav A. Yadav K. (2018). Nanoparticle-based plant disease management: tools for sustainable agriculture. Nanobiotechnology Appl. Plant Prot. 10, 29–39. doi: 10.1007/978-3-319-91161-8_2, PMID: 41841152

[B63] Yusuf A. Almotairy A. R. Z. Henidi H. Alshehri O. Y. Aldughaim M. S. (2023). Nanoparticles as drug delivery systems: a review of the implication of nanoparticles’ physicochemical properties on responses in biological systems. Polymers 15, 3–7. doi: 10.3390/polym15071596, PMID: 37050210 PMC10096782

[B64] Zhang P. Guo Z. Zhang Z. Fu H. White J. C. Lynch I. (2020). Nanomaterial transformation in the soil–plant system: implications for food safety and application in agriculture. Small 16, 3–9. doi: 10.1002/smll.202000705, PMID: 32462786

[B65] Zhou W. Li M. Achal V. (2024). A comprehensive review on environmental and human health impacts of chemical pesticide usage. Emerging Contam. 11, 2–10. doi: 10.1016/j.emcon.2024.100410, PMID: 41842036

